# Ceragenin CSA-13 as free molecules and attached to magnetic nanoparticle surfaces induce caspase-dependent apoptosis in human breast cancer cells via disruption of cell oxidative balance

**DOI:** 10.18632/oncotarget.25105

**Published:** 2018-04-24

**Authors:** Ewelina Piktel, Izabela Prokop, Urszula Wnorowska, Grzegorz Król, Mateusz Cieśluk, Katarzyna Niemirowicz, Paul B. Savage, Robert Bucki

**Affiliations:** ^1^ Department of Microbiological and Nanobiomedical Engineering, Medical University of Bialystok, Mickiewicza 2c, Bialystok 15-222, Poland; ^2^ Department of Medicinal Chemistry, Medical University of Bialystok, Mickiewicza 2d, Bialystok 15-222, Poland; ^3^ Department of Microbiology and Immunology, The Faculty of Health Sciences of the Jan Kochanowski University in Kielce, Aleja IX Wieków Kielc, Kielce 25-317, Poland; ^4^ Department of Chemistry and Biochemistry, Brigham Young University, Provo, Utah 84604, USA

**Keywords:** breast cancer, ceragenin, magnetic nanoparticles, nanotechnology, redox balance

## Abstract

Natural antimicrobial peptides and ceragenins, as non-peptide amphipathic mimics, have been proposed as anti-cancer agents. To date, it has been confirmed that cathelicidin LL-37 and ceragenin CSA-13, both in free form and immobilized on the surface of magnetic nanoparticles (MNP@LL-37, MNP@CSA-13) induce apoptosis in colon cancer cells. Nevertheless, the question remains whether ceragenins, as synthetic analogs of LL-37 peptide and mimicking a number of its properties, act as antineoplastic agents in breast cancer cells, where LL-37 peptide stimulates oncogenesis. Considering potential anticancer activity, we determined whether CSA-13 and MNP@CSA-13 might be effective against breast cancer cells. Our study provides evidence that both CSA-13 and MNP@CSA-13 decreased viability and inhibit proliferation of MCF-7 and MDA-MB-231 cells despite the protumorigenic properties of LL-37 peptide. Flow cytometry-based analyses revealed that ceragenin treatment results in increases in dead and PI-negative/low-viability cells, which was associated with glutathione (GSH) depletion and increased reactive oxygen species (ROS) generation followed by mitochondrial membrane depolarization, caspase activation, and DNA fragmentation. These findings demonstrate that both CSA-13 and MNP@CSA-13 cause disruption of the oxidative balance of cancer cells. This novel mechanism of ceragenin-mediated eradication of cancer cells suggest that these agents may be developed as a possible treatment of breast cancer.

## INTRODUCTION

Breast cancer is one of the most prevalent malignancy that affects women worldwide. According to estimates prepared annually by the American Cancer Society, over 250,000 new cases were expected to be diagnosed in the United States in 2017 [1]. The prevalence of this type of cancer underscores the need for new therapeutic methods and continued analysis of the mechanisms of potential anticancer agents. It is well established that many independent factors such as hormonal therapy, late age of first birth, high body mass index and alcohol use may be considered as risks [2]. However, research conducted over the past few years has shown that development of breast cancer may also be affected by compounds that occur naturally in breast milk and contribute to its anti-infectious properties [3].

Cathelicidin LL-37 belongs to the family of endogenous antimicrobial peptides (AMPs) and is recognized as the only cathelicidin identified in humans [4]. Multiple studies have shown that LL-37 is characterized by a broad spectrum of antimicrobial activity and due to these properties, LL-37 is an important, constitutively-expressed component of innate immunity [3, 5–9]. Additionally, substantial work has shown that LL-37 possesses pleiotropic properties, including the activation of cell proliferation, stimulation of angiogenesis, promotion of wound healing, immunomodulatory activity, and impacts on cancer development [10–12]. However, determination of the precise role in tumorigenesis is strongly impeded due to the varied expression of LL-37 in tumor tissues and its ability to act, both as pro-tumorigenic and anti-cancer agent, depending on the type of cancer [13]. In terms of breast cancer, it was reported that LL-37 increases proliferation of epithelial cells in breast tissues and acts as a putative growth factor, contributing to lymph node metastases in estrogen receptor-positive tumors [14, 15]. At the same time, the usefulness of human cathelicidin in the treatment of cancer diseases has already been demonstrated in combinatory therapy of ovarian cancer; Chuang *et al.* demonstrated that administration of CpG oligodeoxynucleotides (CpG-ODNs) in the presence of LL-37 enhanced anti-cancer activity of CpG-ODNs against ovarian cancer despite the protumorigenic activity of human cathelicidin in ovarian cancer tissues [16].

In contrast to reports demonstrating the varied activity of LL-37 peptide in tumor tissues, ceragenins, as mimics of the human cathelicidin amphipathic properties, have been presented as potential pro-apoptotic compounds in the treatment of cancer [17, 18]. Ceragenins were designed to simulate the facially amphiphilic morphology of antimicrobial peptides with lower costs of production and greater stability under physiological conditions [19]. It is generally accepted that the mechanism of action of ceragenins is due to increases in permeability of the cytoplasmic membranes of pathogens, which is driven by their amphiphilic morphology [20, 21]. Given the above observations, it is suggested that a similar mechanism of action will contribute to the anti-cancer activity of these compounds. To date, results presented by Kuroda *et al.* indicate that ceragenin CSA-13, one of the best studied of the ceragenin group, exerts anti-tumorigenic activity against colon cancer cells through induction of cell cycle arrest followed by intensification of the apoptosis processes [17]. However, it has not yet been determined if ceragenins, as mimics of LL-37 peptide, exert similar anti-tumorigenic activity against cancer cells. A recent study by Olekson *et al.* indicated that ceragenins, including CSA-13, at low concentrations promote human keratinocytes (HaCaT) cell migration and tube formation in an *in vitro* angiogenesis model. It was also suggested that CSA-13 acts through vascular endothelial growth factor receptor 2 (VEGFR2)-mediated pathway, since ZM323881 (i.e. VEGFR2 inhibitor) blocked its formation. Interestingly, CSA-13-induced release of Ca^2+^ was only partially limited by this inhibitor, which imply that CSA-13 acts also by other signaling pathways [22]. Considering these observations, we have studied the potential anti-cancer activity of CSA-13 against breast cancer cells and its mechanism of action.

In recent years, the rapid development of novel nanotechnology-based therapeutic strategies has provided new tools for treatment of malignancies and created the possibility of overcoming limitations of conventional chemotherapy, including low selectivity of chemotherapeutics and associated toxicity against normal host cells. Moreover, the usefulness of nanostructures in the design of drugs with improved pharmacokinetic properties and having the ability to reverse drug resistance of tumors is becoming a focus of research in modern, personalized oncological therapy [23]. With regard to oncological therapy, the increase in the biological activity of anti-cancer drugs in the presence of nanoparticles as drugs carriers is particularly important [24]. Our previous study on colon cancer cells, employing LL-37 peptide and ceragenin CSA-13 immobilized on the surface of iron oxide magnetic nanoparticles, confirmed that AMP-based nanosystems decrease the viability and proliferation ability of cancer cells [18]. However, the mechanism of this phenomenon is still unclear.

Considering the reports described above, we decided to investigate the effects of both ceragenin CSA-13 and its magnetic nanoparticle-based derivative, MNP@CSA-13, on breast cancer cells lines that are known to increase their growth upon stimulation by human cathelicidin LL-37. The mechanism of action of CSA-13 was also analyzed in order to evaluate whether activity of ceragenin-mediated treatment might dependent on different death pathways among various cancer cell lines. Additionally, we performed a series of experiments in order to assess whether the development of a nanosystem based on LL-37 might reverse its protumorigenic effect and increase the effect of ROS-generating MNPs.

Our study provides evidence that both CSA-13 and MNP@CSA-13 should be considered as useful pro-apoptotic agents against breast cancer MCF-7 and MDA-MB-231 cells, leading to disruption of cell oxidative balance followed by induction of caspase-dependent apoptosis and DNA fragmentation. This novel mechanism of ceragenin-mediated anti-cancer activity, different from those previously reported, provides an additional platform for determining their usefulness in the treatment of cancer.

## RESULTS

### Ceragenin CSA-13 and MNP@CSA-13 decrease the viability of human breast cancer cells

**Figure 1:**
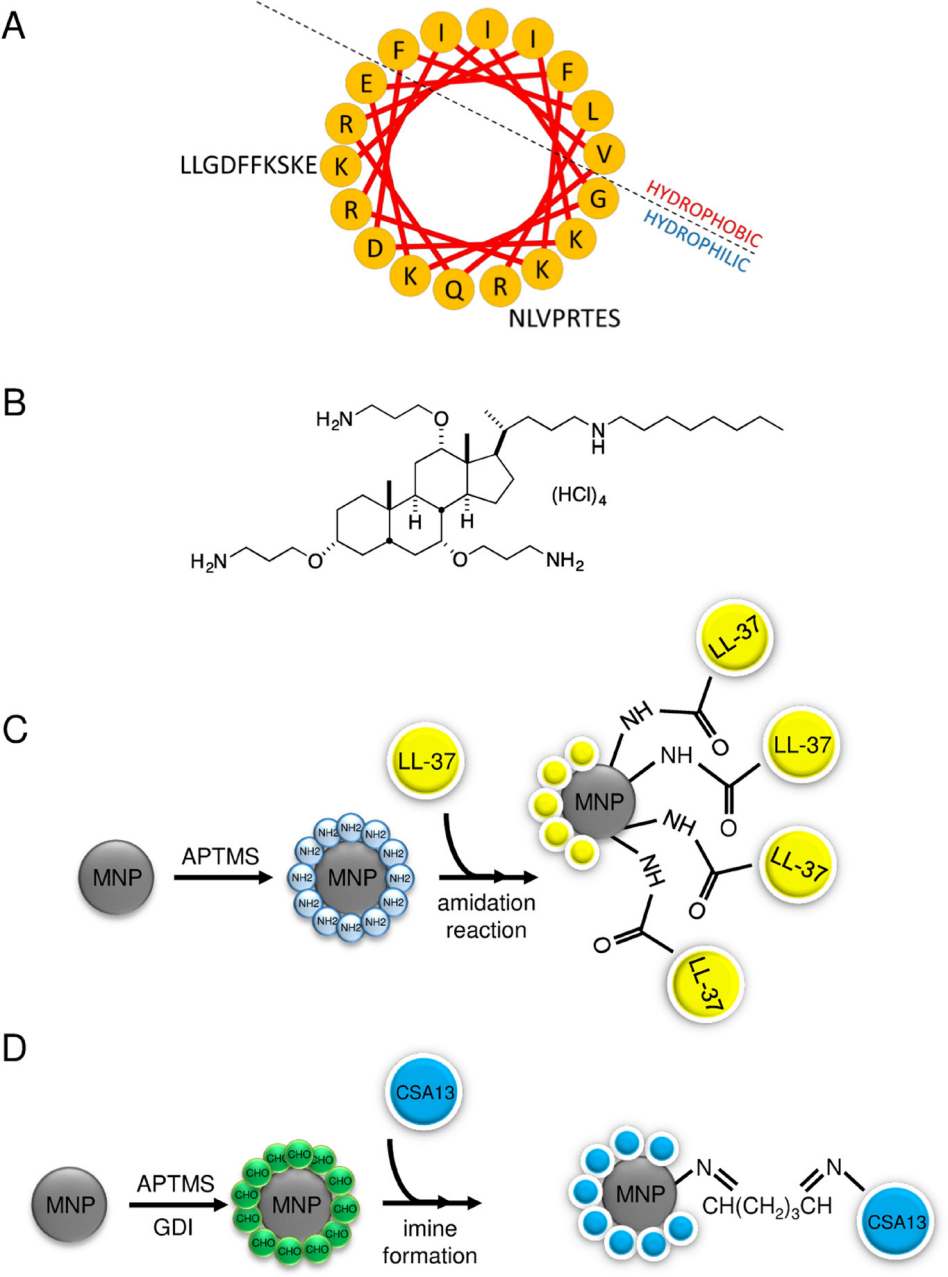
A helix wheel structure of LL-37 peptide (**A**) and structure of ceragenin CSA-13 (**B**). For amino acids, the one-letter code is used. Schematic representation of MNP@LL-37 (**C**) and MNP@CSA-13 (**D**) synthesis.

**Figure 2:**
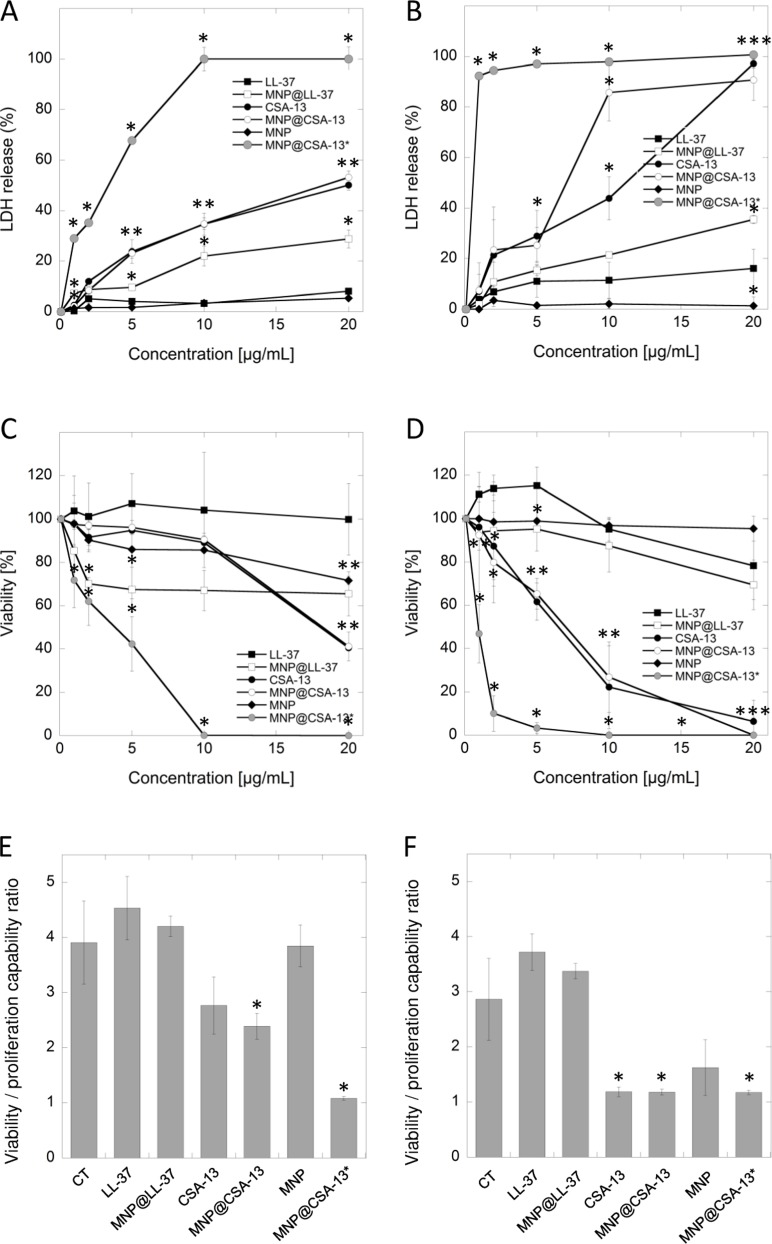
Cytotoxic activity of cationic lipids and their magnetic derivatives against breast cancer MCF-7 (**A**, **C**, **E**) and MDA-MB-231 (**B**, **D**, **F**) cell lines. Increased LDH release from cancer cells (panels A and B) and the decrease of cell viability assessed by MTT assay (panels C and D) after 24 h incubation of cancer cells with varied concentrations of LL-37 peptide (black square), MNP@LL-37 (white square), CSA-13 (black circle), MNP@CSA-13 (white circle) and uncoated MNPs (black diamond). MNP@CSA-13^*^ (grey circle) indicate the alternations in cell viability after treatment with MNP@CSA-13 in doses corresponding to the amounts of CSA-13 immobilized in the nanosystem (assuming the immobilization efficiency of approx. 14%). Panels E and F demonstrate the proliferation of cancer cells treated for 24 hours with 20 µg/mL of tested agents when compared to untreated control (CT; black inverted triangle) estimated using resazurin-based fluorimetric method (panels E and F). Results represent mean ± SD from 3 to 6 independent experiments. ^*^ indicates statistical significance (*p* < 0.05) when compared to LL-37 activity (panels A–D) or untreated control (panels E and F).

In the first stage of the study, the activity of cationic lipids and their magnetic counterparts (Figure 1) against two human breast cancer cell lines, MCF-7 and MDA-MB-21 was assessed. An LDH release assay was employed, and the agents were used at concentrations ranging from 1 to 20 µg/mL, because higher doses of ceragenins resulted in the lysis of cells and were toxic against normal healthy cells (data not shown). Cancer cells were treated in serum-free conditions for 24 hours, since serum-starvation lasting longer than 24 hours considerably affected the viability of cells (Supplementary Figure 1A). At the same time, treatment lasting 24 hours did not affect results; viability of ceragenin-treated cells did not differ when measured using serum-free and serum-containing media (Supplementary Figure 1B). As presented in the Figure 2A and 2B, ceragenin CSA-13 alone and immobilized on the surface of magnetic nanocarrier at the dose of 20 µg/mL strongly increased the release of LDH from treated cancer cells resulting in the killing of 50.15 ± 2.02% and 53.18 ± 2.50% of MCF-7 cells and 97.20 ± 1.75% and 90.74 ± 8.01% of MDA-MB-231 cells, respectively. In contrast to these results, uncoated MNPs had no significant effect on the viability of treated cells. These results were confirmed using the MTT assay; after treatment of cells with CSA-13 and MNP@CSA-13 at a dose of 20 µg/mL, only 40.14 ± 1.02% and 41.14 ± 6.58% of MCF-7 cells were able to undertake proper metabolic activity (Figure 2C). MDA-MB-231 cells were more sensitive to this synthetic analog, with almost no detectable metabolism observed in these samples (Figure 2D). These data were confirmed using a resazurin-based viability/proliferation capability assay. As demonstrated in the Figure 2E and 2F, CSA-13 and MNP@CSA-13 effectively limited the proliferation capability of cancer cells.

In addition to the results obtained during the treatment of cancer cells using CSA-13 and MNP@CSA-13 at doses ranging from 1 to 20 µg/mL, we compared the activity of CSA-13 and MNP@CSA-13 in doses corresponding to the amounts of CSA-13 immobilized in the nanosystem. According to our earlier assessment, the immobilization efficiency of the nanosystem synthesis process is approx. 14% [25]. Considering this efficiency, we measured the viability of cancer cells after treatment with agents containing a comparable amount of ceragenin. As expected, the viability of cancer cells incubated with the nanosystem was significantly reduced, resulting in the eradication of MCF-7 and MDA-MB-231 cells, at doses of 10 and 20 µg/mL, respectively, which strongly indicates that immobilization of ceragenin CSA-13 on the surface of magnetic nanocarrier strongly improves its biological activity resulting in stronger anti-cancer activity.

### Immobilization of LL-37 on the surface of magnetic nanoparticles does not significantly affect its protumorigenic activity

One of the theories guiding this research is that functionalization of magnetic nanoparticles possessing well-known antineoplastic activity [18] with LL-37, characterized by considerable membrane-permeabilizing properties and diverse activity against various cancer tissues [13] would result in the creation of nanosystem inhibiting the viability of cancer cells, despite the protumorigenic activity of human cathelicidin. Nevertheless, fluorimetric-based analysis revealed that immobilization of LL-37 peptide on MNPs indeed caused a decline in the protumorigenic activity of cathelicidin, but this effect was too weak to demonstrate anti-cancer properties of such a combination since the proliferation ratio for MNP@LL-37 was still stronger than for untreated control samples (Figure 2E and 2F). Moreover, additional analyses including the measurement of key apoptotic features such as DNA fragmentation or externalization of phosphatidylserine did not reveal statistically significant differences between LL-37 alone and in combination with MNPs, despite the increased intracellular accumulation of MNP@LL-37 when compared to the unimmobilized form (Supplementary Figure 2). We suggest that intracellular uptake of these structures is responsible for the anti-cancer activity of MNP@LL-37. Nevertheless, the anti-cancer effect of this nanoformulation was relatively weak, and no further analyses of this nanosystem against breast cancer cells were performed.

### The high accumulation of CSA-13 and MNP@CSA-13 assures their anti-cancer activity against breast cancer MCF-7 cells

**Figure 3:**
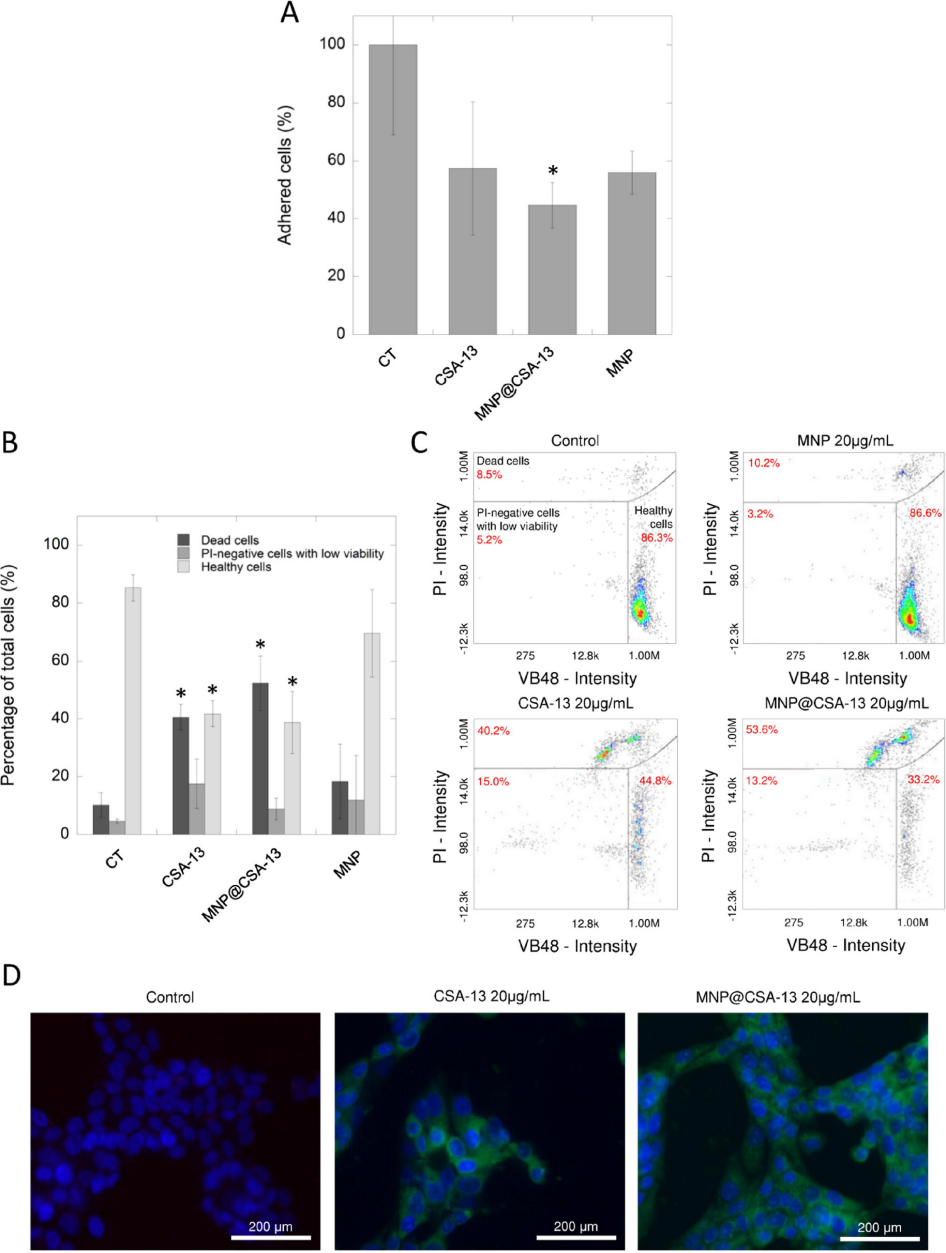
The impact of CSA-13 and MNP@CSA-13 on viability and adhesion of treated MCF-7 cells The adhesion of MCF-7-treated cells to the cell-treated surface assessed using CV staining (**A**). The percentages of dead cells (dark grey bars), PI-negative cells with low viability (grey bars) and healthy cells (light grey bars) in MCF-7 cells treated with CSA-13, MNP@CSA-13 and naked MNPs (**B**). (**C**) shows representative plots from flow cytometry analysis of viability of ceragenin-treated cells. Intracellular uptake of FITC-labeled CSA-13 and MNP@CSA-13 is demonstrated in the (**D**). All experiments were performed using agents at concentration of 20 µg/mL for 24 h. Panels A and B present results from 3 independent experiments ± SD, for panels C and D results from one representative experiment were shown. ^*^indicates statistical significance (*p* < 0.05) when compared to untreated control (panel A) or corresponding cells population in control samples (panel B).

In contrast to results obtained with MNP@LL-37, additional colorimetric- and flow cytometry-based analyses performed using the MCF-7 cell line confirmed preliminary results indicating the possibility of eradicating cancer cells using ceragenin-based agents. Considering the data reported in the first stage of the study, we chose the dose of 20 µg/mL for further analysis. As presented in the Figure 3A, the number of cells attached to the cell-treated surface after treatment with CSA-13 and MNP@CSA-13 were reduced to 57.39 ± 23.04% and 44.69 ± 7.82%, respectively. Moreover, the double staining of ceragenin-treated cells with VB-48 and propidium iodide (PI) revealed that a number of dead and PI-negative cells with low viability was 3.94- and 4.15-fold higher for samples treated with CSA-13 and MNP@CSA-13 respectively when compared to untreated control and nearly 2-fold higher in comparison to samples treated with MNPs alone (Figure 3B and 3C). To understand the enhanced effect of agents immobilized on the surface of magnetic nanoparticles, a fluorescence microscope-based analysis, in which breast cancer cells were treated with CSA-13 as free molecules and in immobilized form, was performed. For this purpose, both CSA-13 and MNP@CSA-13 were labeled with FITC and visualized using fluorescence microscopy. Results of this analysis are shown in Figure 3D. As shown, both agents accumulated in treated cancer cells with considerably stronger effects noted for MNP@CSA-13, which is proposed as an explanation of enhanced anti-tumorigenic activity of this nanoagent when compared to its non-magnetic counterpart.

### Treatment of breast cancer with CSA-13 and MNP@CSA-13 induces apoptosis

**Figure 4:**
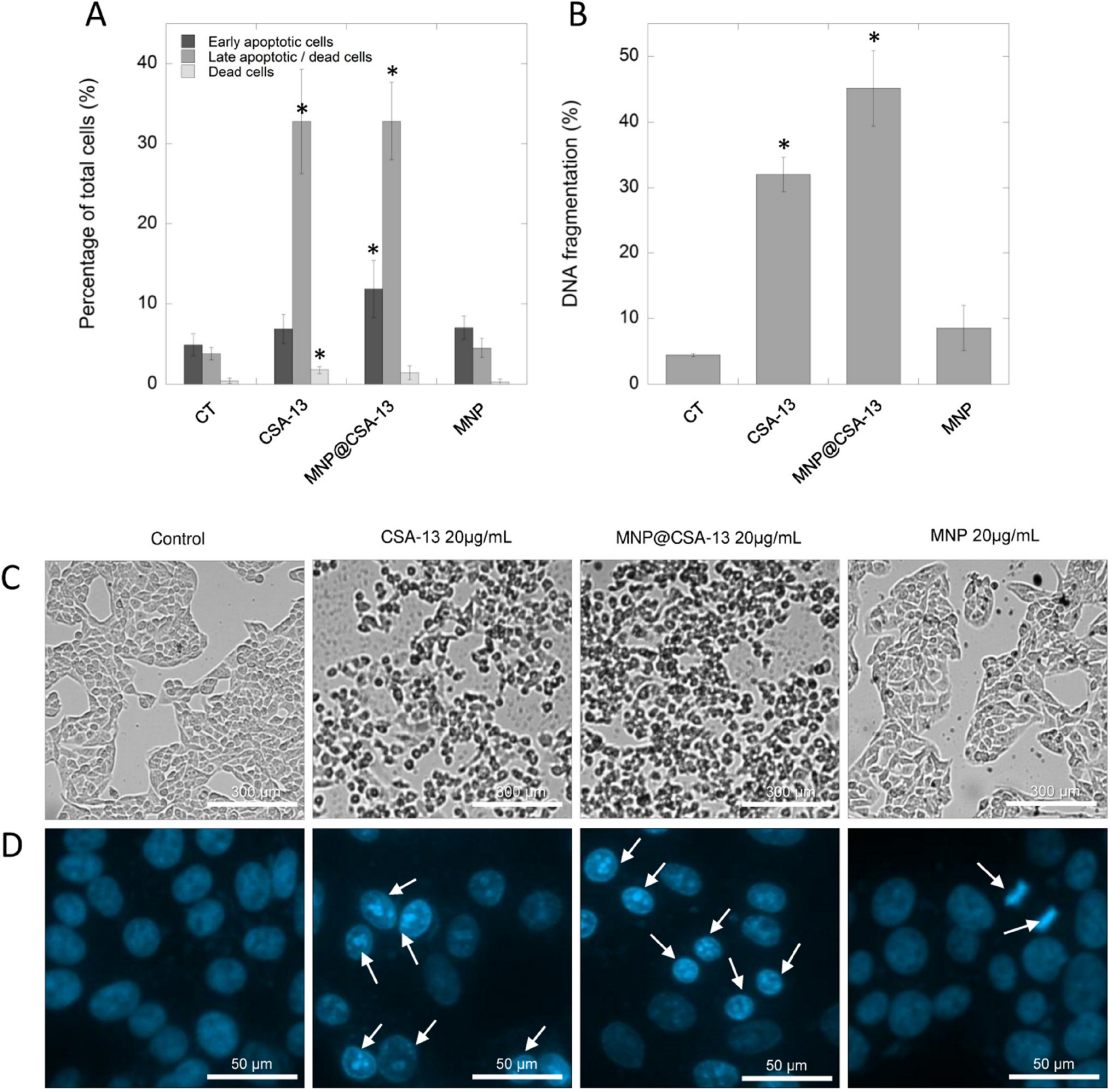
Induction of apoptosis and DNA fragmentation in MCF-7 cells by CSA-13 and its magnetic derivative Percentage of early apoptotic (dark grey bars), late apoptotic/dead cells (grey bars) and dead cells (light bars) (**A**) and the level of DNA fragmentation (**B**) in MCF-7 cells after ceragenin-mediated treatment. Panel C demonstrates alternations in morphology of treated cancer cells assesses using phase contrast microscopy, panel D shows morphological alternations in nuclei of MCF-7 cells upon treatment with CSA-13, MNP@CSA-13 and MNPs when compared to uncreated cells. White arrows indicate treatment-induced morphological changes in nuclei. All experiments were performed using agents in a concentration of 20 µg/mL for 24 h. Panels A and B present results from 3 independent experiments ± SD, for panels (**C**) and (**D**) results from one representative experiment were shown. ^*^indicates statistical significance (*p* < 0.05) when compared to corresponding cells population in control samples (panel A) or untreated control (panel B).

Previous data reported by Kuroda *et al.* using CSA-13 as an anti-neoplastic agent against colon HCT116 cancer cells indicated that ceragenin-treated cells underwent apoptosis-mediated cell death [17]. In order to evaluate the mechanism of CSA-13- and MNP@CSA-13-induced killing of breast cancer cells, MCF-7 cells were analyzed by flow cytometry using Annexin V-FITC/7-AAD double staining. A combination of Annexin V and 7-AAD allowed the cells to be sorted into four groups: early apoptotic cells [Annexin V(+)/7-AAD(–)], late apoptotic/dead cells [Annexin V(+)/7-AAD(+)], dead cells [Annexin V(–)/7-AAD(+)], and live cells [Annexin V(–)/7-AAD(–)]. The calculated percentages of apoptotic and dead cells are presented in the Figure 4A. As demonstrated, after 24 h of incubation with 20 µg/mL of CSA-13 and MNP@CSA-13, a significant number of cells were in late stage apoptosis with both CSA-13 (32.80 ± 6.53%, *p* = 0.0016) and its magnetic derivative (32.82 ± 4.82%, *p* = 0.0005) (Supplementary Figure 3). However, it was noted that some of these cells were still undergoing the early apoptosis process, since 6.90 ± 1.81% and 11.90 ± 3.57% of cells, respectively, were classified as Annexin V-positive/7-AAD-negative. Nevertheless, these results clearly indicate that ceragenin-based treatment induces apoptosis in breast cancer cells. Importantly, this process occurs very fast, since first indicators of apoptosis are recognized after an eight-hour treatment of cancer cells with the indicated agents (Supplementary Figure 4A).

One of the key apoptotic features in individual cells is fragmentation of DNA, observed as the result of the activity of calcium and magnesium-dependent nucleases degrading DNA and causing nicks and double-stand breaks in the genetic material of treated cells [26]. This late apoptosis event was detected by a flow cytometry method employing DAPI, a DNA-binding dye. As presented in Figure 4B, CSA-13 and its magnetic derivative are potent inducers of DNA fragmentation in MCF-7 breast cancer cells causing fragmentation of genetic material in 32.00 ± 2.59% and 45.13 ± 5.73% of treated cancer cells, respectively, which significantly affected cell morphology (Figure 4C). These results were confirmed using fluorescence microscopy (Figure 4D); in contrast to control samples characterized by round nuclei with homogeneous chromatin and exhibiting a weaker blue color, CSA-13 and MNP@CSA-13-treated cell nuclei showed the classical morphological characteristics of apoptosis, i.e. reduction in nuclear size, chromatin condensation, and DNA fragmentation.

### The anti-cancer activity of CSA-13 and MNP@CSA-13 is determined by disruption of GSH redox status and caspase-dependent apoptosis

**Figure 5:**
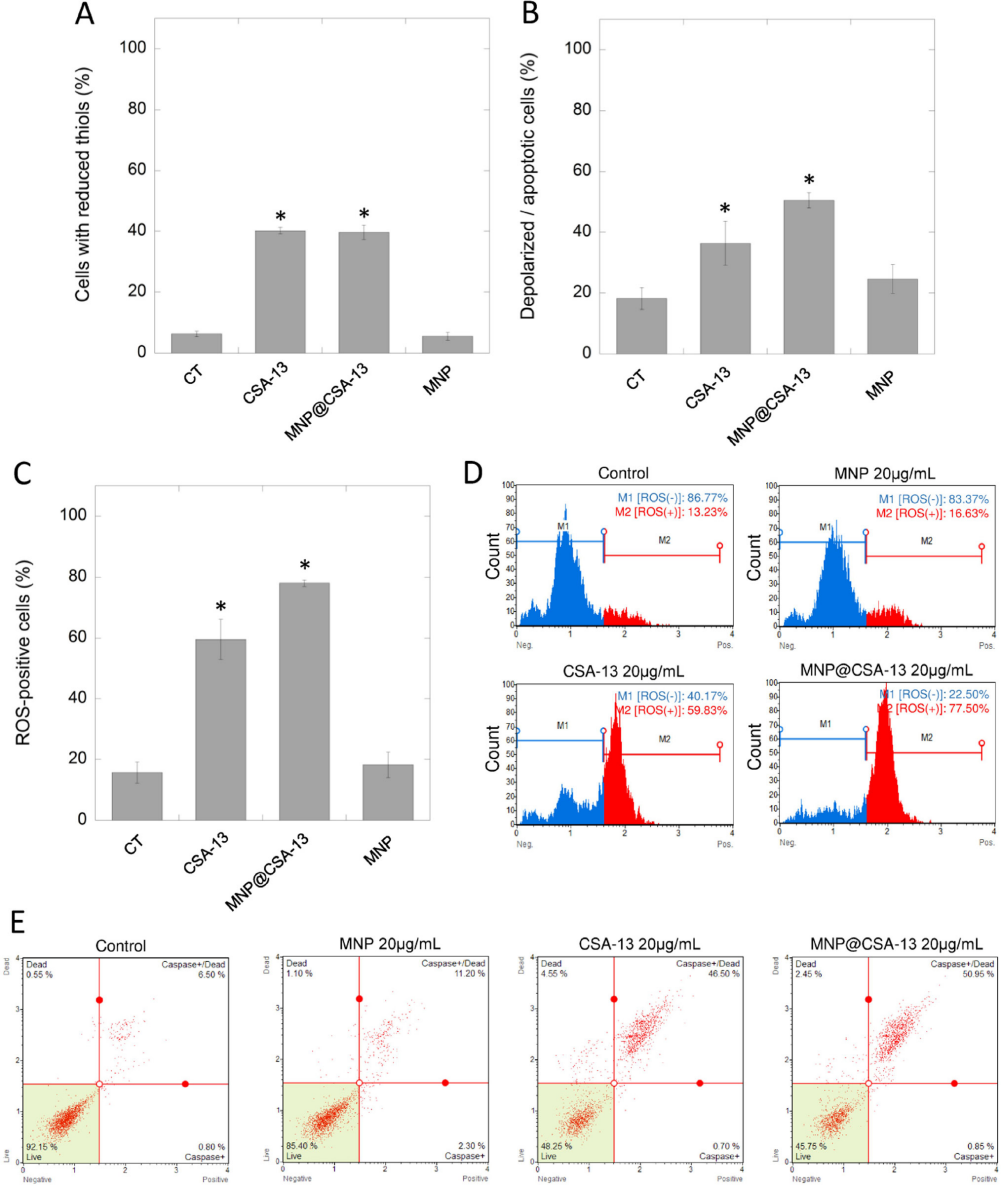
CSA-13 and MNP@CSA-13 disrupt the oxidative balance in treated cancer cells and induce mitochondrial pathway of apoptosis A number of ceragenin-treated cells with reduced thiols and cells with depolarized mitochondria is presented in the panel (**A**) and (**B**), respectively. Panel (**C**) shows percentages of ROS-positive cells as assessed using flow cytometry measurement. Representative plots from ROS generation assay are provided in the panel (**D**). Blue part of plot indicates ROS-negative cells (M1), red part presents ROS-positive cells (M2). Panel E demonstrates representative plots from caspases activity measurements. All experiment were performed using agents in a concentration of 20 µg/mL for 24 h. Panels (A–C) present results from 3 independent experiments ± SD, for panels (D and E) results from one representative experiment were shown. ^*^indicates statistical significance (*p* < 0.05) when compared to untreated control (panel A).

In order to investigate the mechanism of antineoplastic activity of CSA-13 and its magnetic derivative, we performed a series of flow cytometry-based experiments focused on the evaluation of ceragenin-mediated alternations in the oxidative balance of treated cancer cells. As a result of ceragenin-mediated cell treatment, we detected significant decline in cellular antioxidant glutathione (GSH) levels using VB-48 as the indicator of intracellular thiol levels. Incubation (24 h) of breast cells with both CSA-13 and MNP@CSA-13 significantly intensified the reduction of GSH and increased the number of cells with reduced thiols to 40.15 ± 1.06% and 39.67 ± 2.35% (*p* < 0.0001), which is a 6.4-fold increase when compared to untreated control (Figure 5A).

Considering reports indicating that the decrease of cellular GSH below a threshold level initiates mitochondrial apoptotic signaling and that the mitochondrial membrane potential of cells undergoing the apoptosis process is typically lost, we performed an analysis of the mitochondrial potential of treated cancer cells. As expected, significant numbers of cells characterized by mitochondrial membrane depolarization were detected (36.33 ± 7.25% [*p* = 0.0176] and 50.50 ± 2.55% [*p* = 0.0002] for CSA-13 and MNP@CSA-13, respectively), which indicated increased mitochondrial injury in treated cells when compared with control samples (Figure 5B, Supplementary Figure 5). These results suggested that ceragenin-mediated cell death could be associated with an imbalance in oxidative status of treated cells. To evaluate this, DHE-based flow cytometry assay was performed. The results of this analysis are shown in the Figures 5C and 5D. Presented data indicate that exposure of cells to CSA-13 and MNP@CSA-13 resulted in increases in levels of reactive oxygen species in a time-dependent manner (Supplementary Figure 4B). This effect was significantly higher for MNP@CSA-13 than for CSA-13 alone (78.03 ± 1.04% compared to 59.54 ± 6.57%, *p* value = 0.0086), which is possibly related to increased intracellular accumulation of this agent and additional ROS-generating ability of uncoated MNPs. Moreover, investigation of caspase activity using a Fluorescent-Labeled Inhibitor of Caspases (FLICA)-based assay confirmed that ceragenin-mediated killing of cancer cells is caused by the activity of caspases (Figure 5E). The level of necrotic cells (caspase-negative and 7-AAD-positive) was also enhanced in CSA-13 and MNP@CSA-13-treated samples (4.601 ± 1.126% and 2.92 ± 0.534% vs 0.5167 ± 0.416% in control samples), which might suggest that additional mechanisms are engaged in ceragenin-mediated cell death; however, this increase seemed to be insignificant when compared to the level of caspases-positive cells.

## DISCUSSION

Despite the constant development of novel anti-cancer therapies, breast cancer remains the most common and one of the most lethal cancers among women worldwide, with mortality of 14% [1]. Among a variety of established risk factors predisposing women to the development of breast tumors, reports demonstrating the protumorigenic activity of naturally occurring proteins seems to be the most important. Such a factor is the LL-37 peptide, the only member of the natural antimicrobial peptides from the cathelicidin family identified in humans [10]. A variety of studies suggests that the impact of LL-37 on tumor development is determined by the origin of the cancer tissue and by the ability to bind different membrane receptors whose expression varies on different cancer cells. Human cathelicidin exerts both inhibitory and protumorigenic activity against cancer tumors [13, 27]. Studies performed by Armogida *et al.* showed that breast milk cells expressed mRNA for a number of mediators of the innate immune system, including LL-37 peptide, which clearly indicates that human cathelicidin is one of the key factors constituting an innate human defense system of the mammary gland epithelium in the human breast [3]. Nevertheless, despite the protective role of this peptide in human milk, it has been demonstrated that human cathelicidin acts as a growth factor for breast epithelial cells, and its overexpression contributes to lymph node metastases in estrogen receptor-positive tumors [14, 15]. Our results generated using colorimetric- and fluorimetric-based methods are in the agreement with these reports indicating that LL-37 peptide increases metabolic activity (Figure 2C and 2D) of both MCF-7 and MDA-MB-231 cells, particularly at doses ranging from 1 to 10 µg/mL and promotes the proliferation of LL-37-treated cells (Figure 2E and 2F) confirming the protumorigenic activity of this peptide in breast cancer cell cultures.

In contrast to the data demonstrating the ability of human cathelicidin to induce cancer development in some cell types, ceragenins are proposed as potential anti-cancer agents [19]. Ceragenins were designed as mimics of endogenous antimicrobial peptides, reproducing the facially their amphiphilic morphology but with simpler methods of production and greater stability under physiological conditions. To date, it has been reported that ceragenin CSA-13 and LL-37-derived peptides, FK-16 and FF/CAP18, inhibit colon cancer progression due to initiation of caspase-independent apoptosis in cancer cells and induction of cell cycle arrest [17, 28, 29]. Additionally, our recent study on colon cancer cell lines DLD-1 and HCT116 showed that viability of cancer cells after treatment with LL-37 and CSA-13 immobilized on the surface of magnetic nanoparticles is significantly reduced [18].

Considering the above-described reports, we decided (i) to evaluate whether immobilization of LL-37 peptide on the magnetic surface might alter the biological activity of LL-37 peptide from protumorigenic to anti-neoplastic, (ii) to assess if cathelicidin-mimicking ceragenin CSA-13 and its magnetic counterpart, MNP@CSA-13 composed of MNPs functionalized with CSA-13, exert anti-tumorigenic activity against breast cancer cells, and (iii) to investigate whether possible anti-cancer activity is determined by the same mechanism of action, as presented previously for colon cancer cells. In our previous research, focused mainly on the evaluation of antimicrobial activity of this compound, we confirmed the efficient immobilization of CSA-13 on the surface of aminosilane-coated iron oxide nanoparticles and satisfactory stability of this nanosystem in experimental settings [18, 25].

In the first stage of this study, we determined that attachment of human cathelicidin on the magnetic nano-carrier is insufficient to obtain a nanosystem exerting activity against breast cancer cells. Despite the promising results obtained in LDH and MTT-based assays (Figure 2) showing a decrease of breast cancer cell viability after treatment with MNP@LL-37, studies with more sensitive fluorimetric- and flow cytometry-based methods indicated minimal anti-cancer activity (Supplementary Figure 2). Viability assays using resazurin revealed that MNP@LL-37-treated cells are characterized by greater viability and proliferation capability than untreated controls (Figure 2E and 2F). Moreover, the MNP@LL-37-mediated increase of caspase activity or DNA fragmentation when compared to unimmobilized peptide and naked MNPs was not statistically significant (Supplementary Figure 2). We hypothesize that partial shifting of the LL-37-mediated activity from promoting to inhibiting the growth of cancer cells resulted from increased uptake and intracellular accumulation of ROS-generating nanoparticles, which would be in agreement with previous reports indicating that human cathelicidin is able to increase the uptake and activity of antineoplastic molecules co-administered simultaneously, even in cells whose growth should be stimulated by LL-37, such as ovarian cancer cells [16]. However, the cellular effects obtained in the result of this process are still too weak to confirm the anti-cancer activity of this combination.

In contrast to unsatisfactory results obtained for MNP@LL-37, it was demonstrated that ceragenin CSA-13 and its magnetic derivative, MNP@CSA-13, might be potent tools in the eradication of breast cancer cells. Previously, Weber *et al.* demonstrated that mRNA expression of hCAP18/LL-37 in breast cancer cells is strongly correlated with the presence of lymph node metastasis in estrogen receptor-positive tumors from clinical samples. On the other hand, no significant difference in hCAP18 levels with respect to lymph node status was observed for ER-negative patients, which indicates that protumorigenic activity of LL-37 in breast cancer cells is linked with ER status [14]. Considering this, we decided to evaluate the cytotoxic activity of CSA-13 in both, ER-positive and ER-negative breast cancer cell lines. Despite the differences in ER status and the protumorigenic properties of LL-37 peptide in breast cancer cells, we demonstrated that viability and proliferation ability of both MCF-7 and MDA-MB-231 is significantly reduced in the presence of CSA-13 and MNP@CSA-13 (Figure 2). Particularly high anti-cancer effectiveness was noted for MDA-MB-231 since a dose of 10 µg/mL of MNP@CSA-13 was sufficient to eradicate the majority of cancer cells. We suggest that higher sensitivity of this breast cancer cell line was determined by its phenotype; the MDA-MB-231 cell line is classified as triple-negative (estrogen receptor/progesterone receptor/HER2–negative) cell line and is recognized as more prone to cytotoxic treatment because of its lack of DNA repairing capability [30]. Importantly, the killing effect of ceragenin-mediated treatment was observed at relatively low doses of CSA-13 and MNP@CSA-13 (10–20 µg/mL) which are recognized as non-toxic when assessed using an hemolysis assay, as described previously by Niemirowicz *et al.* [25]. An important observation noted formerly by our research team was the fact that immobilization of ceragenin on the surface of nano-carrier significantly improves its biocompatibility, which would be a great advantage in the design of non-toxic nanotechnology-based anti-cancer therapies [23]. In contrast to CSA-13, whose high doses ranging from 50 to 100 µg/mL cause hemolysis of red blood cells as a consequence of its membrane activity, MNP@CSA-13 did not affect red blood cell membrane permeability at a concentration range of 1 – 100 μg/ml [25]. Additionally, an important advantage of MNP@CSA-13 over unimmobilized ceragenin is also the increased cytotoxic effect observed during the course of our study. Double VB-48/PI staining provided data about MNP@CSA-13-induced increased population of dead/low viability cells when compared to CSA-13 alone (Figure 3B and 3C). A higher level of DNA fragmentation (Figure 4B), mitochondrial membrane depolarization (Figure 5B, Supplementary Figure 5), ROS generation (Figure 5C, Supplementary Figure 4B) and caspase-positive cells (Figure 5E) also confirmed that immobilization of CSA-13 on the surface of magnetic nanoparticles might provide improvements in the anti-cancer efficiency of these agents.

The main aim of this study was to investigate the potential mechanism of killing breast cancer cells using ceragenin CSA-13 and its magnetic counterpart. Previous studies performed by Kuroda *et al.* demonstrated that CSA-13 decreases the viability and proliferation of HCT116 colon cancer cells by apoptosis-mediated cell death [17]. Niemirowicz *et al.* confirmed that a similar mechanism is responsible for the eradication of HT-29 and DLD-1 colon cancer cells by CSA-13 and MNP@CSA-13 [18]. Our results are in the agreement with these reports; as presented in the Figure 4A and Supplementary Figure 4A both CSA-13 and MNP@CSA-13-treated breast cancer cells underwent apoptosis after 24 h incubation with indicated agents as evaluated by double cell staining with FITC-Annexin V/7-AAD. This conclusion was further supported by the observation of DNA fragmentation, being the key event of the late apoptosis process and other apoptosis-related features including mitochondrial membrane depolarization and GSH depletion, which is considered an early hallmark in the progression of apoptosis in response to difference stimuli (Figure 5A and 5B, Supplementary Figure 5) [31].

It is generally accepted that glutathione (GSH) is the most abundant intracellular regulator of cell redox status involved a variety of detoxification mechanisms and the protection of cells against ROS generation and followed by it oxidative-mediated damage and cell death [32]. Correlation between cellular GSH depletion and the induction of apoptosis has been established, which clearly indicates that maintaining of the balance of a redox environment is vital for the survival of the cell [31]. This phenomenon is further supported by reports indicating that the depletion of intracellular glutathione affects the apoptosis process by predisposing the cells to death via sensitizing the cell to apoptotic stimuli or by promoting mitochondrial permeabilization [33, 34]. Moreover, Franco *et al.* reported that GSH depletion is necessary to induce the apoptosis process activated by both extrinsic and intrinsic signaling pathways [35]. In contrast, higher levels of GSH in cells have been related to apoptosis resistance [36]. Godwin *et al.* reported that enhanced resistance of ovarian cancer cells lines to cisplatin is associated with increased levels of cellular glutathione [37]. Cazanave *et al.* demonstrated as well that increased cellular GSH levels constitute a protection against Fas-induced apoptosis [38].

Many chemotherapeutics affect the cellular redox status; for example, Alemany *et al.* reported that the anti-neoplastic effect of arsenic trioxide (As_2_O_3_) against human megakaryocytic leukemia cell lines is achieved via multiple cellular effects, including modification of the glutathione redox system [39]. The anti-cancer activity of alantolactone against three human glioblastoma cell lines U87, U373, and LN229 is caused by GSH depletion followed by ROS generation and mitochondrial dysfunction as reported by Khan *et al.* [40]. The same research team has reported that alantolactone-induced apoptosis in hepatocellular carcinoma HepG2 cells is determined by depletion of GSH, which results from the direct conjugation of alantolactone with this antioxidant [41]. These reports are in agreement with data demonstrating that GSH depletors such as buthionine sulphoximine (BSO) and diethylmaleate (DEM) enhance the cytotoxicity cis-dichlorodiammineplatinum (CDDP) through an augmentation of ROS generation in bladder cancer cells [42].

The decrease of intracellular concentration of GSH was also noted by our research team in ceragenin-treated breast cancer cells. Flow cytometry analysis clearly showed that cellular level of GSH is strongly depleted by both CSA-13 and MNP@CSA-13 indicating the strong effect of ceragenin-mediated treatment on the redox status of treated cells (Figure 5A). Simultaneously, we observed increased levels of intracellular ROS, which is in the agreement with previous studies demonstrating that anti-neoplastic agents acting through GSH depletion affect the overproduction of ROS at the same time (Figure 5C, Supplementary Figure 4B) [40, 41]. Interestingly, both CSA-13 and MNPs exert ROS-promoting ability that suggests a reason for enhanced cytotoxic activity of the ceragenin-based nanosystem. The next stages of our analysis were influenced by reports showing that disruption of cellular redox balance and GSH depletion promote the mitochondrial pathway of apoptotic signaling by triggering mitochondrial depolarization [31, 43]. As expected, the number of cells characterized by the loss of mitochondrial membrane potential increased significantly after CSA-13- and MNP@CSA-13-mediated treatment (Figure 5B, Supplementary Figure 5), which confirms the connection between imbalance of redox status and depolarization of mitochondrial membrane. Nevertheless, it should also be noted that this effect might be furthermore induced by membrane-permeabilizing properties of ceragenins. To date, it is established that the mechanism of AMP-mediated antimicrobial killing involves binding to the external surfaces of negatively charged cytoplasmic bacterial membranes and inducing pore formation in cellular membranes, which leads to the leakage of cytoplasmic content and cell death [44]. Considering reports indicating that the eukaryotic mitochondrial membrane is structurally similar to microbial membranes, having a high content of anionic phospholipids, it is suggested that the diffusion of the peptide into the cells through insertion into the cellular membrane allows for the subsequent interaction with the mitochondrial membrane [45]. In agreement with this hypothesis, it was reported that some highly cationic antimicrobial peptides, including BMAP-28, a bovine antimicrobial peptide of the cathelicidin family, induce apoptosis in tumor cells through opening of the mitochondrial permeability transition pore [46]. Given these observations, we suggest that CSA-13-induced mitochondrial depolarization might results from both membrane activity of the ceragenin and its impact on the redox status of breast cancer cells.

The FLICA-based analysis confirmed that ceragenin-induced apoptosis is controlled by the activity of caspases (Figure 5E). This is consistent with studies presented previously by Jia *et al.* reporting that apoptosis induced by tubeimoside-1, a natural compound isolated from tubeimoside, is caused by the enhancement of ROS causing the release of cytochrome c and activation of caspase-3 [47]. The release of cytochrome c from cancer cells was also noted during the investigation of alantolactone-mediated treatment of glioma cells [40].

Interestingly, the observed mechanism of ceragenin-mediated killing of cancer cells is considerably different from those reported for earlier research by Kuroda *et al.* [17]. It was previously demonstrated that treatment of colon cancer HCT116 cells with CSA-13 results in the inhibition of cell viability and proliferation, but through a caspase-independent mechanism, since no mitochondrial membrane depolarization, caspase activity and DNA fragmentation were observed during the course of the study [17]. In contrast to these findings, all of these apoptotic features were noted in our experimental settings. We propose that the differences in the mechanism of ceragenin-mediated treatment could be determined by the employment of a different cell line that might affect the course of biological activity of ceragenin and its magnetic derivative.

Considering the studies described above and our results, we propose that both CSA-13 and MNP@CSA-13 possess the potential to be used as potent anti-cancer tools in the treatment of breast cancer. These materials cause induction of cell death, via disruption of cell oxidative balance, with subsequent mitochondrial depolarization and release of caspases, DNA damage, and fragmentation. This novel mechanism of ceragenin-mediated anti-cancer activity, different from those previously reported, suggests that activity of AMP-based analogs might vary between cancer cell lines. Nevertheless, according to the presented data it is assumed that CSA-13 exerts an opposite effect from LL-37 peptide, causing a decrease in the viability of breast cancer cells. Importantly, the activity of MNP@CSA-13 and its unique physicochemical properties established by the immobilization of ceragenin on the surface of a magnetic nanocarrier provide the possibility to improve the biocompatibility of CSA-13 and to employ this agent in the controlled and personalized therapy of breast tumors.

## MATERIALS AND METHODS

### Synthesis of LL-37- and CSA-13-decorated nanosystems

The LL-37 peptide was synthesized and provided by Lipopharm.pl (Zblewo, Poland). According to HPLC analysis provided by the manufacturer, the purity of synthetized peptide was >98%. The helix-wheel structure of LL-37 peptide is presented in the Figure 1A. A representative of ceragenins, CSA-13 (Figure 1B), was synthesized as described previously [48]. Immobilization of LL-37 peptide and CSA-13 onto the nanoparticle surface was achieved by an amidation reaction in the case of LL-37 or formation of imine bonds in the case of ceragenin between an amine group and the MNP surface [18, 25]. Physicochemical properties of synthesized nanostructures were analyzed using transmission electron microscopy (TEM), Fourier transform infrared spectroscopy (FT-IR), differential scanning calorimetry (DSC) and thermogravimetric analysis (TGA). Details concerning the synthesis of MNP@LL-37 and MNP@CSA-13 and their physicochemical characterization were presented in the earlier research [25]. The summary of nanosystem preparation is presented in the Figure 1C and 1D. The loading efficiency of LL-37 and CSA-13 on the surface of magnetic nanoparticles were calculated based on thermogravimetric analysis, which indicated the immobilization of agents on the MNPs with ∼ 25% and ∼ 14% LL-37 and CSA-13 content, respectively [25, 49].

### Cell culture

Human breast cancer cell lines MCF-7 and MDA-MB-231 were a gift from the collection of Department of Medicinal Chemistry (Medical University of Bialystok, Poland). The cultures were grown in high-glucose DMEM (Dulbecco′s Modified Eagle′s Medium) supplemented with 10% fetal bovine serum (FBS), glutamine (2 mM/L), penicillin (50 U/mL) and streptomycin (50 µg/mL). The cells were maintained at 37° C in an atmosphere containing 5% CO_2_ with saturated humidity. After seeding of cells, the experiments were performed in serum-free conditions in order to avoid factors affecting the results for indicated time. As presented in the Supplementary Figure 1B, no differences in the viability of ceragenin-treated cells were observed when using serum-free and serum-containing media for 24 hours.

### Evaluation of cytotoxicity activity of tested agents against MCF-7 and MDA-MB-231 cell lines

The viability of breast cancer MCF-7 and MDA-MB-231 cells after incubation with tested agents was assessed by measurement of release of lactate dehydrogenase (LDH) from treated cells according to the protocol provided in the manufacture’s manual. In order to evaluate the cytotoxicity of tested agents, the cells were seeded at a density of 5 × 10^3^ cells per well in 200 µL of growth medium in 96-well plates. Analyzed agents were added at concentrations ranging from 1 to 20 µg/mL for 24 hours. Results are presented as percent cell viability compared to untreated control, while cells treated with lytic solution were considered as 100% LDH release. To confirm results, the viability and metabolic activity of treated cancer cells were measured using microculture tetrazolium test (MTT; 3-(4,5-dimethylthiazol-2-yl)-2,5-diphenyltetrazolium bromide) as described previously [50]. The absorbance value obtained in cultures of control cells (without tested agents) was taken as 100%. The average of all the experiments has been shown as cell viability percentage in comparison to the control. In order to evaluate the impact of cationic lipids and their magnetic derivatives (10µg/mL) on cell proliferation capability, the resazurin-based fluorimetric assay was performed [51].

### Adherence assay

The adherence of MCF-7 cells upon drug treatment was assessed by staining of pre-fixed attached cancer cells with crystal violet staining solution (0.5%) according to the protocol presented by Feoktistova *et al.* [52].

### Uptake of the FITC-labeled agent into cancer cells

Internalization of CSA-13 and its magnetic derivative was evaluated using fluorescent microscopy (BD Pathway Bioimaging Systems, BD Biosciences, San Jose, CA, USA). Labeling of tested compounds with fluorescein isothiocyanate (FITC) was performed as previously described [53]. MCF-7 cells were seeded in 96-well culture plates at the density of 5 × 10^3^ cells per well before the tested agents were added to the final concentration of 20 µg/mL. The cell nuclei were visualized by staining with Hoechst 33342 dye. Images were collected at 400× magnification.

### Annexin V staining

The externalization of phosphatidylserine (PS) to the cell surface in the response to the treatment with CSA-13 and its magnetic analog (20 µg/mL) was measured using Muse^®^ Annexin V & Dead Cell Kit (Merck, Germany). The combination of FITC-labeled Annexin V with 7-AAD (7-aminoactinomycin D; indicator of cell membrane structural integrity) allows distinguishing the early and late apoptotic cells. For the purpose of the clarity of presented data, live cells (Annexin V-negative and 7-AAD-negative) were not presented in the provided figures.

### Evaluation of DNA fragmentation and alternations in nuclei morphology

Detection of alternations in DNA content in treated breast cancer MCF-7 cells was performed using a DNA Fragmentation Assay Kit prepared for NucleoCounter^®^ NC-3000™ system (ChemoMetec, Denmark), following the manufacturer’s instructions. MCF-7 cells were treated with tested agents at the dose of 20 µg/mL for 24 hours, harvested, washed and fixed in 70% ethanol for 12 hours. Ethanol-suspended cells were centrifuged for 5 min at 500 g, resuspended in Solution 3 containing 1 µg/ml DAPI and 0.1% triton X-100 in PBS, loaded onto an NC-Slide and DNA content histograms were collected. To visualize the alternations in nuclei morphology after exposure of cells to CSA-13, MNP@CSA-13 and uncoated MNPs, apoptotic cells with condensed and fragmented nuclei was examined under a fluorescence microscope (BD Pathway Bioimaging Systems, BD Biosciences, San Jose, CA, USA). For this purpose, cells were seeded at the density of 5 × 10^3^ cells/mL in 96 well plates, cultured for 24 hours and treated with tested agents at a dose of 20 µg/mL. Then, cells were washed phosphate buffered saline (PBS) and fixed with 4% paraformaldehyde in PBS for 15 min at room temperature. After incubation with 0.1% Triton X-100 in PBS (5 min, room temperature), cells were thoroughly washed and stained with DAPI solution (1 µg/mL) for 5 min at room temperature. Results from one representative experiment are presented.

### Vitality and GSH evaluation assay

In order to assess the intracellular concentration of GSH, Vitality assay was engaged, employing fluorescent dye VitaBright-48™ (VB-48), which reacts with thiols forming a fluorescent product (ChemoMetec, Denmark). In order to additionally assess the viability of treated cancer cells, samples were stained with acridine orange (AO) and propidium iodide (PI), and cell populations were measured by analysis of VB-48™ intensity versus the intensity of PI. For this purpose, MCF-7 cells were treated with 20 µg/mL of CSA-13, MNP@CSA-13 and MNPs for 24 hours. Cells were then washed with sterile PBS, harvested and incubated with Solution 5 composed of the mixture of VB-48, propidium iodide and acridine orange.

### Evaluation of ROS generation

Flow cytometry-based quantitative measurement of reactive oxygen species (ROS) in cells undergoing oxidative stress was performed using Muse^®^ Oxidative Stress Kit prepared for Muse Cell Analyzer (Merck, Germany), according to the protocol provided in manufacture’s manual. Muse^®^ Oxidative Stress Reagent is based on dihydroethidium (DHE), which is recognized as a well-known reagent in the detection of ROS in cell cultures [54]. For the purpose of the experiment, cells were treated with 20 µg/mL of CSA-13, MNP@CSA-13 and MNPs for 24 hours.

### Evaluation of mitochondrial potential

The detection of alternations in mitochondrial potential was detected using Mitochondrial potential assay (ChemoMetec, Denmark), following the manufacturer’s instructions. In this assay, MCF-7 cells were treated with tested agents at the concentration of 20 µg/mL for 24 hours, washed, harvested and incubated with 2.5 µg/mL of JC-1 for 20 minutes. Then, cells were thoroughly washed with PBS and added to a solution of DAPI in PBS (1µg/mL) in order to stain necrotic and late apoptotic cells. Prepared samples were immediately analyzed using NucleoCounter^®^ NC-3000™ system.

### Multicaspase activity assay

Muse^®^ MultiCaspase Assay Kit (Merck, Germany) was engaged in order to quantify the number of cells with caspase activity. This assay was based on the employment of Fluorescent-Labeled Inhibitor of Caspases (FLICA) allowing for the detection of the presence of activity of multiple caspases (caspase-1, 3, 4, 5, 6, 7, 8, and 9). In this set of experiments breast, cancer MCF-7 cells were treated with tested agents for 24 hours at the dose of 20 µg/mL. For the purpose of the clarity of presented data, live cells with no detected caspase activity (FLICA-negative and 7-AAD-negative) were not presented in the provided figures.

### Statistical analysis

Provided data are results from 3 to 6 independent experiments ± SD. Significance of differences was determined using the two-tailed Student’s *t*-test. Statistical analyses were performed using Statistica 10 (StatSoft Inc, Tulsa, OK, USA). *P* < 0.05 was considered to be statistically significant.

## SUPPLEMENTARY MATERIALS FIGURES



## Abbreviations

7-AAD: 7-aminoactinomycin D
AMPs: antimicrobial peptides
CSAs: cationic steroid antibiotics
CV: crystal violet
DAPI: 2-(4-amidinophenyl)-1H -indole-6-carboxamidine
DHE: dihydroethidium
DMEM: Dulbecco′s Modified Eagle′s Medium
PBS: phosphate buffered saline
FBS: fetal bovine serum
FITC: fluorescein isothiocyanate
FK-16: a fragment of LL-37 corresponding to residues 17 to 32
FLICA: Fluorescent-Labeled Inhibitor of Caspases
FF/CAP18: 27-residue analog of the LL-37 peptide
GSH: glutathione
LDH: lactate dehydrogenase
LL-37: cathelicidin delivered 37 amino acid peptide
MNPs: magnetic nanoparticles
MNP@CSA-13: CSA-13 attached to magnetic nanoparticles
MNP@LL-37: LL-37 attached to magnetic nanoparticles
MTT: 3-(4,5-dimethylthiazol-2-yl)-2,5-diphenyltetrazolium bromide
PI: propidium iodide
ROS: reactive oxygen species
