# Supplementary material for: Ceragenin CSA-13 as free molecules and attached to magnetic nanoparticle surfaces induce caspase-dependent apoptosis in human breast cancer cells via disruption of cell oxidative balance
Source: Oncotarget. 2018 Apr 24;9(31):21904–20. doi: 10.18632/oncotarget.25105 (PMC5955147; doi:10.18632/oncotarget.25105)
Supplement: Supplementary file 1 [file oncotarget-09-21904-s001.pdf]

Ceragenin CSA-13 as free molecules and attached to magnetic nanoparticle surfaces induce caspase-dependent apoptosis in human breast cancer cells via disruption of cell oxidative balance

SUPPLEMENTARY MATERIALS

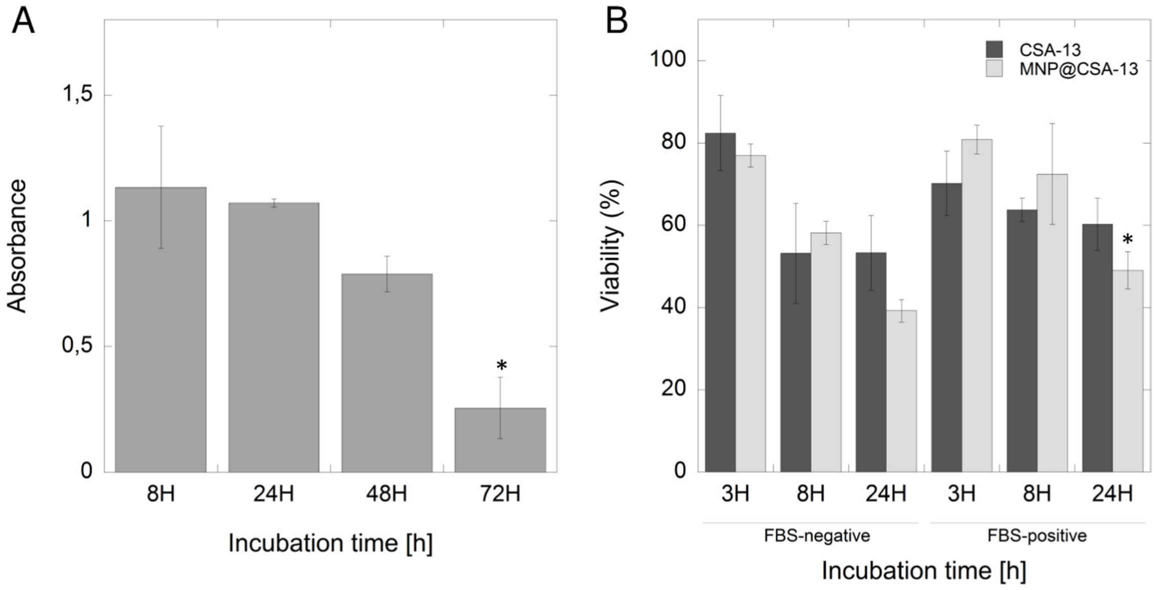

Supplementary Figure 1: The time-dependent decrease of MCF-7 cells viability in the absence of fetal bovine serum (FBS) (**A**). The viability of MCF-7 cells after treatment with CSA-13 and MNP@CSA-13 for 3, 8 and 24 h in the absence or presence of FBS (**B**). Results from 3 independent experiments ± SD are presented. *indicates statistical significance ($p < 0.05$) when compared to viability of cells after 8 hour incubation (A) or corresponding cells population in FBS-negative samples (B).

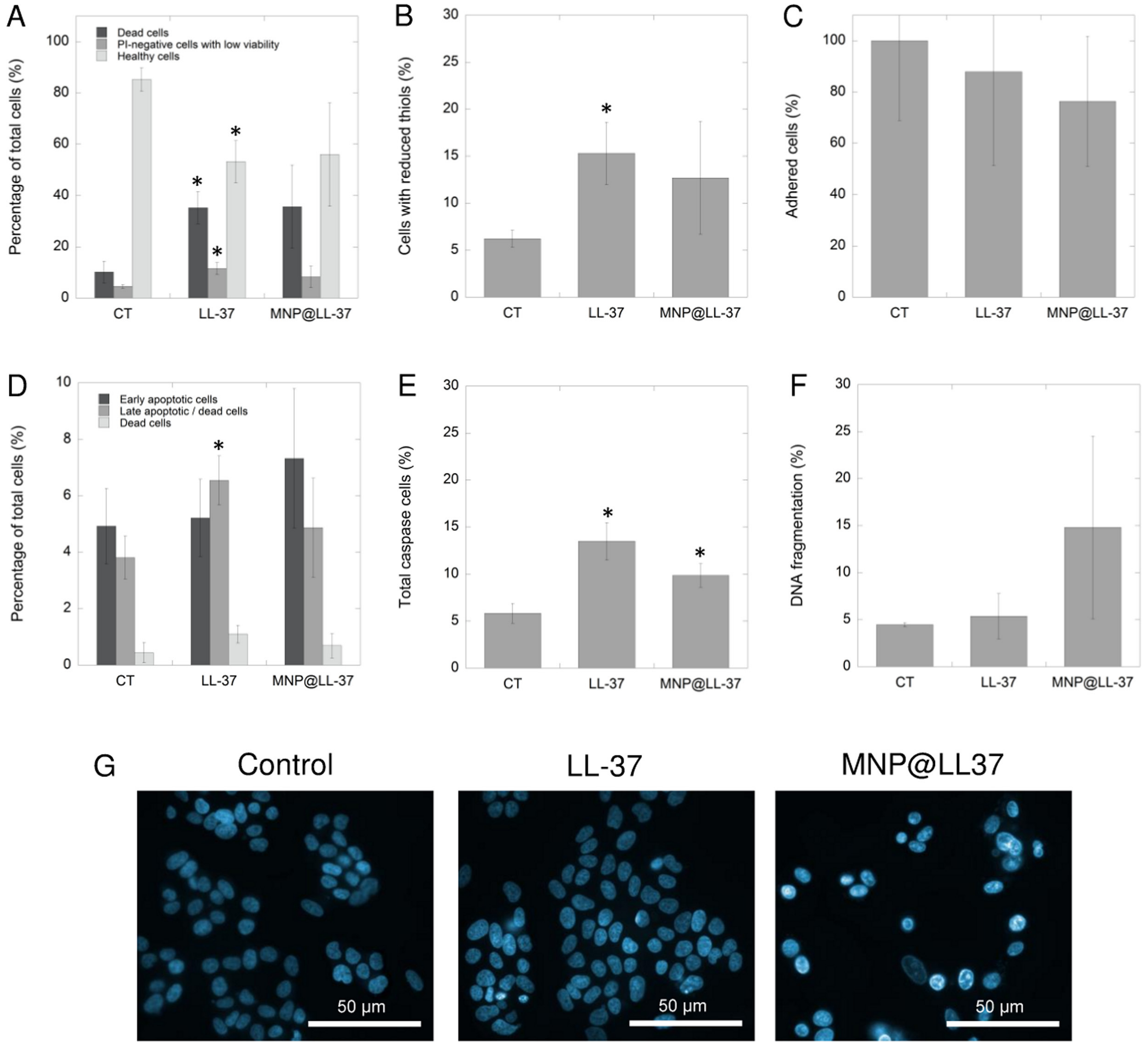

Supplementary Figure 2: The activity of LL-37 peptide and its magnetic derivative, MNP@LL-37 against MCF-7 cells. The impact of LL-37/MNP@LL-37 on viability (**A**), intracellular GSH level (**B**) and adhesion (**C**) of treated MCF-7 cells. Panels (**D**–**E**) demonstrate the level of apoptosis and caspase activation in treated cancer cells, respectively. The level of DNA fragmentation and alternations in nuclei morphology after treatment with indciated agents is presented in the panels (**F** and **G**). All experiments were performed using agents at concentration of 20 µg/mL for 24 h. Panels (A –F) present results from 3 independent experiments ± SD, for panel G results from one representative experiment were shown. *indicates statistical significance ($p < 0.05$) when compared to corresponding cells population of untreated control (CT; panels A, D) or untreated control (CT; panels B, C, E, F).

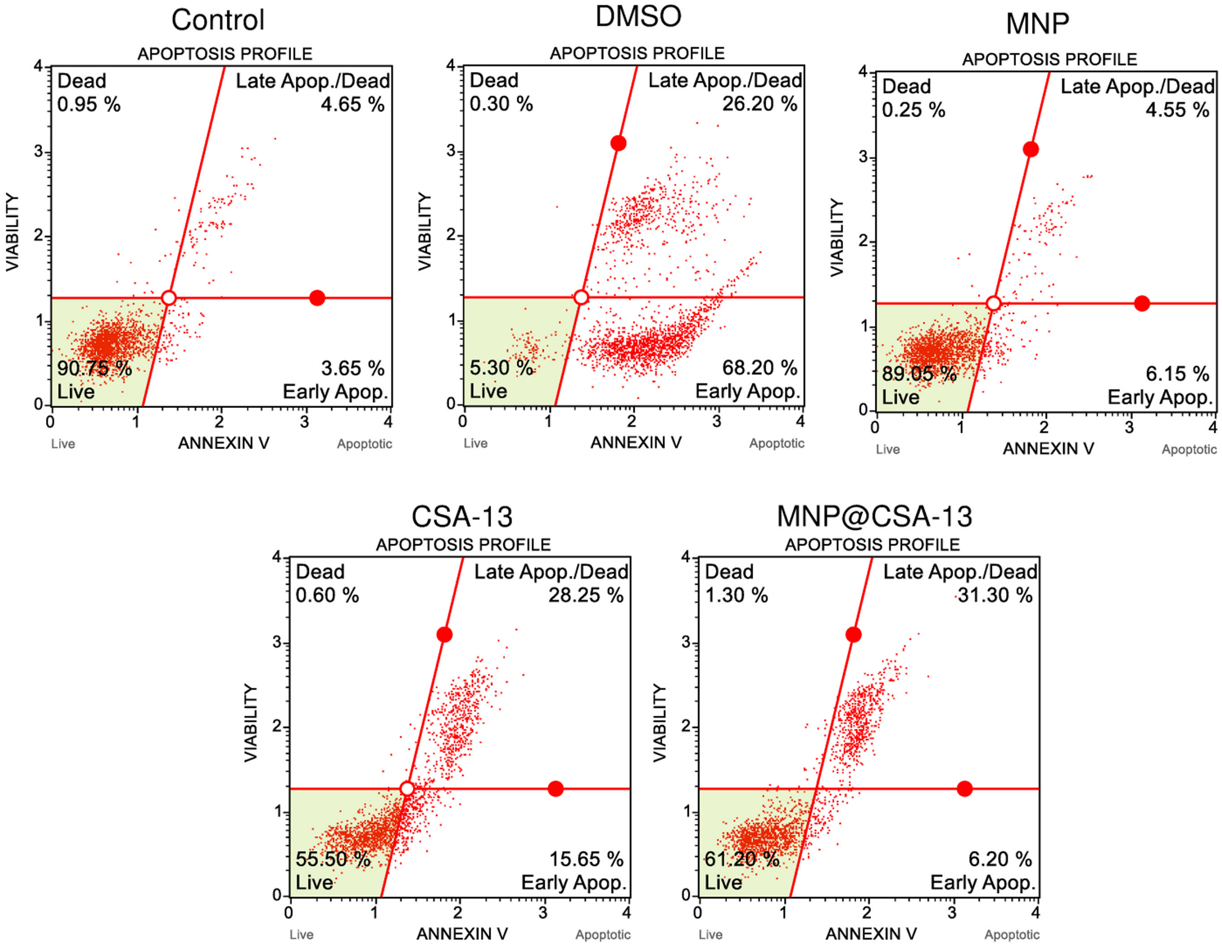

Supplementary Figure 3: The representative plots of cytometry flow analyses of MCF-7 cells treated with CSA-13/ MNP@CSA-13 at the dose of 20 µg/mL for 24 h followed by Annexin V-FITC and PI staining.

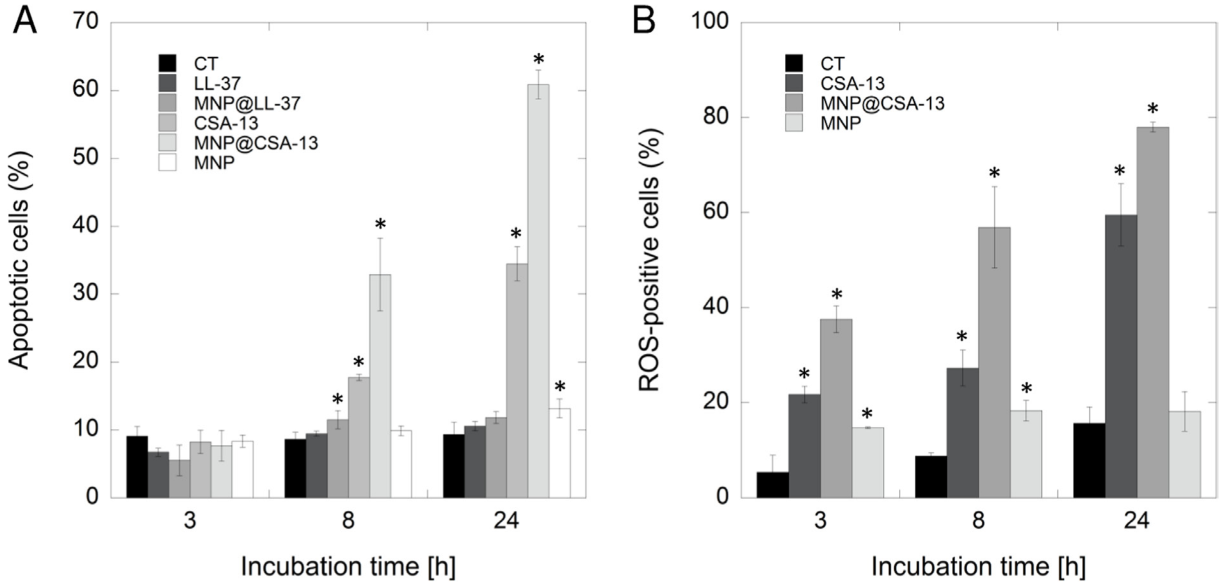

Supplementary Figure 4: The kinetic of apoptosis (**A**) and ROS formation induction (**B**) by cationic lipids (LL-37, CSA-13) and their magnetive derivative (MNP@LL-37, MNP@CSA-13) assessed using flow cytometry method. Results from 3 independent experiments ± SD are presented. *indicates statistical significance ($p < 0.05$) when compared to untreated control (CT).

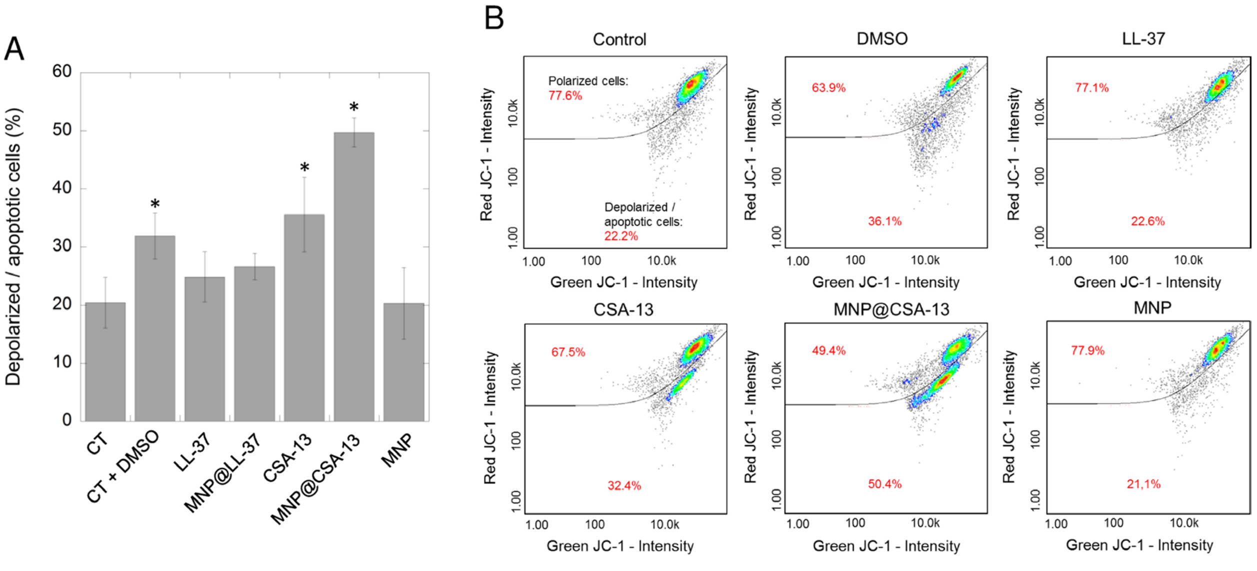

Supplementary Figure 5: The impact of cationic lipids (LL-37, CSA-13) and their magnetic derivatives (MNP@LL-37, MNP@CSA-13) on mitochondrial potential of MCF-7 cells treated with indicated agents at the dose of 20 µg/mL for 24 h. The summary of results obtained from 6 independent experiments ± SD are presented in (**A**) Representative plots of flow cytometry analysis are demonstrated in (**B**). *indicates statistical significance ($p<0.05$) when compared to untreated control (CT).
